# Clinical prediction model for tumor progression in Barrett’s esophagus

**DOI:** 10.1007/s00464-018-6590-5

**Published:** 2018-11-19

**Authors:** Dag Holmberg, Eivind Ness-Jensen, Fredrik Mattsson, Jesper Lagergren

**Affiliations:** 1 Upper Gastrointestinal Surgery, Department of Molecular Medicine and Surgery, Karolinska Institutet, Karolinska University Hospital, Norra Stationsgatan 67, Solna (L1:00), 171 76 Stockholm, Sweden; 2 Department of Public Health and Nursing, HUNT Research Centre, NTNU, Norwegian University of Science and Technology, Forskningsvegen 2, 7600 Levanger, Norway; 3 Department of Medicine, Levanger Hospital, Nord-Trøndelag Hospital Trust, P.O. Box 333, 7601 Levanger, Norway; 4 School of Cancer Sciences, King’s College London, New Hunt’s House, Guy’s Campus, London, SE1 1UL UK

**Keywords:** Esophageal adenocarcinoma, Barrett’s esophagus, Surveillance, Endoscopy, High-grade dysplasia

## Abstract

**Background:**

Individuals with Barrett’s esophagus (BE) are at increased risk of high-grade dysplasia (HGD) and esophageal adenocarcinoma (EAC), but the cost-effectiveness of general surveillance of BE is low. This study aimed to identify a risk prediction model for tumor progression in individuals with BE based on age, sex, and risk factors found at upper endoscopy, enabling tailored surveillance.

**Methods:**

This nested case–control study originated from a cohort of 8171 adults diagnosed with BE in 2006–2013 in the Swedish Patient Registry. Cases had EAC/HGD (*n* = 279) as identified from the Swedish Cancer Registry, whereas controls had no EAC/HGD (*n* = 1089). Findings from endoscopy and histopathology reports were extracted from medical records at 71 Swedish hospitals and from the Swedish Patient Registry. Multivariable logistic regression provided odds ratios (OR) with 95% confidence intervals (CIs).

**Results:**

Older age (OR 1.02 [95% CI 1.01–1.03] per year), male sex (OR 2.8 [95% CI 1.9–4.1]), and increasing maximum BE length (OR 2.3 [95% CI 1.4–3.9] for segments 3–8 cm and OR 4.3 [95% CI 2.5–7.2] for segments ≥ 8 cm) increased the risk of EAC/HGD, while the circumferential extent of the BE, hiatal hernia or reflux esophagitis did not. A model based on age, sex, and maximum BE length predicted 71% of all EAC/HGD cases.

**Conclusions:**

A simple combination of the variables age, sex and maximum BE length showed fairly good accuracy for predicting tumor progression in BE. This clinical risk prediction model may help to tailor future surveillance programs.

**Electronic supplementary material:**

The online version of this article (10.1007/s00464-018-6590-5) contains supplementary material, which is available to authorized users.

The incidence of esophageal adenocarcinoma (EAC) has increased sixfold since the 1970s, and it is now the predominant subtype of esophageal cancer in the United States, Europe, and Australia [1]. EAC develops through a well-defined pathway, triggered by pathologic reflux of duodeno-gastric contents to the lower esophagus, which in turn induces metaplasia, i.e., Barrett’s esophagus (BE), followed by high-grade dysplasia and invasive EAC. BE occurs in 1–2% of adults in European populations [2, 3], and individuals with BE retain a tenfold increased risk of EAC compared to the background population [4, 5]. Patients diagnosed with EAC have an overall 5-year survival below 25%, mainly due to late presenting symptoms and detection at advanced stages [6]. Based on the premise that early-stage EAC detection reduces mortality, individuals with known BE are often continuously surveyed by upper endoscopy. However, given that the incidence rate to EAC is only 1–4 cases per 1000 person-year at risk [4, 5], general surveillance of all individuals with BE is not cost-effective [7, 8]. Thus, there is a need to tailor surveillance programs to include individuals with BE at high absolute risk of tumor progression. Risk prediction modeling may help endoscopists or other physicians to estimate the individual’s risk of tumor progression and select patients with BE for tailored surveillance or no surveillance, depending on the individual patient’s risk factor profile. An ideal risk prediction model would consist of a few, easily identifiable variables, and still accurately discriminate between individuals at high and low risk. Older age, male sex, and increasing maximum extent of the segment might increase the risk of tumor progression, whereas the etiologic role of circumferential extent of the segment, hiatal hernia, and reflux esophagitis is less studied [9]. The aim of the present study was to reveal a clinically useful risk prediction model for tumor progression in BE based on age, sex, and endoscopic variables by means of a large case–control study nested within an unselected cohort of individuals with BE.

## Materials and methods

### Study design

This was a Swedish nationwide case–control study nested within a cohort of individuals with a confirmed BE diagnosis between January 1, 2006 (when K22.7 was introduced in Sweden) and December 31, 2013 (end of the study period). All cohort members with BE were identified by searching the Swedish Patient Registry for the diagnosis code for BE (K22.7) in the International Classification of Diseases version 10 (ICD-10), either as a main or secondary diagnosis. A diagnosis of BE in Sweden requires presence of the characteristic BE lesion upon endoscopy combined with a histological examination showing specialized intestinal metaplasia. Cases were those in the BE cohort who developed EAC or HGD, identified from the Swedish Cancer Registry based on the ICD-7 codes for cancer of the esophagus (150) or gastroesophageal junction (151.1) with histopathology (C24.1 Histology Code) codes for adenocarcinoma (096) or HGD (094). Individuals with BE who had incident or prevalent EAC/HGD were eligible as cases, based on the premise that the clinical and demographic features in these two groups would be similar. For each case of EAC/HGD, four controls were randomly identified among all BE cohort members without EAC/HGD. Three endoscopic variables were evaluated as potential risk factors for tumor progression: length of the BE segment, hiatal hernia, and reflux esophagitis. Information about these variables was extracted from medical records and the Swedish Patient Registry. Other potential risk factors for tumor progression, such as obesity and history of smoking, were not assessed because data on these factors were not routinely documented in the endoscopy reports. The study was approved by the Regional Ethical Review Board in Stockholm, Sweden (diary number 2013/1267-31/2, September 18, 2013).

### Data collection

*The Swedish Patient Registry* provided data for collecting the source BE cohort, as well as the participants’ age, sex, date of diagnosis, department and hospital diagnosing the BE, and the presence of hiatal hernia and reflux esophagitis diagnoses. This registry contains information about time and place of diagnoses according to the ICD classification from all in-patient and specialized out-patient health care in Sweden from 2001 onwards. The Patient Registry has been validated for its excellent usefulness for research purposes, with a positive predictive value of any primary diagnosis of 85–95%, which further increases to 90–98% when there is an associated procedure to detect the diagnosis, e.g., endoscopy [10].

*The Swedish Cancer Registry* was used to identify all cases of EAC and HGD. This registry was started in 1958, and records all newly diagnosed malignancies in Sweden according to a standardized formula, including data on tumor site, stage, and histological type. The completeness of EAC registration in the Cancer Registry is 98% [11].

*The Swedish Prescribed Drug Registry* provided data on use of proton pump inhibitors. The registry was established on July 1, 2005, and contains information on all prescribed and dispensed medications in Sweden. The collection of data is automatized through computer-based systems, making the registration almost 100% complete [12].

Registration to the above three registries is mandatory by law for Swedish healthcare, which contributes to the high completeness.

*Endoscopy and histopathology reports* were requested for the cases and controls from all 80 hospitals in Sweden that diagnosed the individuals with BE in the cohort. For cases, both endoscopies from the date of the BE diagnosis and the EAC/HGD diagnosis were requested. In instances where the retrieved index endoscopy was non-descriptive, incomplete, or unavailable, additional records were requested. One author (D.H.) reviewed the endoscopy and histopathology reports and recorded the information into a database, all according to a pre-defined study protocol. Three endoscopic variables were assessed, as detailed below.

1. *The extent of the BE segment* was assessed according to the Prague criteria in centimeters (cm) of circumferential (C) and maximum (M) segment length [13]. Whenever the segment length was described as the distance in cm’s from the dental arch, the average distance from the dental arch to the gastroesophageal junction was assumed to be 40 cm in men and 38 cm in women, unless otherwise specified in the endoscopy report. The maximum BE length was categorized into four groups: segments lengths of < 1 cm were defined as “ultra-short,” 1 to < 3 cm as “short,” 3 to < 8 cm as “long,” and ≥ 8 cm as “ultra-long.” If the length of the BE segment was described in less detail in the endoscopy report, e.g., as “short” or “long,” it was included into the most suitable of the four categories. Fragmented Z-lines or isolated BE islands were classified as ultra-short BE.
2. *Hiatal hernia* was defined as present or absent, and when present the axial length was determined from the endoscopy. An axial length of 1 to 2 cm was categorized as “small,” > 2 to < 5 cm as “medium,” and ≥ 5 cm as a “large” hernia. If the size of the hernia was described more vividly, e.g., in comparison to fruits, the diameter of the specific fruits was approximated to a corresponding axial length. Additionally, the Swedish Patient Registry was searched for the ICD-10 code for hiatal hernia (K44), either as a main or secondary diagnosis. If a hiatal hernia diagnosis was recorded in the Patient Registry, but not reported in the endoscopy, it was recorded as the presence of hiatal hernia with missing data for size.
3. *Reflux esophagitis* was determined as present or absent and whenever present, the severity was assessed from the endoscopy reports according to the Los Angeles criteria [14]. Additionally, the ICD-10 code for reflux esophagitis (K21.0), registered as main or secondary diagnosis at any point in time in the Patient Registry, was used to assess any esophagitis not reported at endoscopy. Patients with a reflux esophagitis diagnosis without information about the Los Angeles criteria were recorded as having esophagitis with missing data for severity.

The unique ten-digit personal identity number, assigned to all individuals permanently residing in Sweden, enabled accurate cross-linking of each individual’s data between registries and the medical data collection.

### Statistical analysis

The statistical analyses were conducted according to a detailed protocol formed upon study inception. Logistic regression was used to calculate odds ratios (OR) with 95% confidence intervals (CI) for the association between the endoscopic variables and the binary outcomes (EAC/HGD or not). Both unadjusted and adjusted models were conducted testing the following five variables: sex (male or female), age in years at the diagnosis of BE (continuous), maximum BE length (< 1, ≤ 1 to < 3, ≤ 3 to < 8, or ≥ 8 cm), hiatal hernia (yes or no), and reflux esophagitis (yes or no). Three binary outcomes were assessed in separate models: combined EAC/HGD, EAC only, and HGD only. For categorical variables, the assumed lowest risk category was used as the reference, e.g., ultra-short BE length. In the adjusted model, adjustments were made for age, sex, and for the other endoscopic variables using the same categorization as presented above. First-order interactions were evaluated for all variables using the likelihood ratio test, with no statistical significant interactions defined by a 5% level of significance. Analyses evaluating effect modification were conducted for the combined EAC/HGD outcome. A receiver operating characteristic curve was fitted to assess the accuracy of predictions. To manage partial missing information on maximum BE length (10%), multiple imputation analysis was performed in addition to the complete case analysis. The number of imputed data sets was 20 and monotone logistic method in PROC MI was used with the assumption that the missing data were missing at random [15]. The variables included in the imputation were sex, age, maximum Barrett length, hiatal hernia and esophagitis with the same categorization as presented above. PROC MIANALYZE was used to combine the results from the analyses of the 20 datasets. Goodness-of-fit of the final prediction model was assessed by the Hosmer and Lemeshow test. A senior biostatistician (F.M.) conducted all statistical analysis and data management using the statistical software SAS Statistical Package (version 9.4, SAS Institute Inc., Gary, NC).

## Results

### Patients

**Fig. 1:**
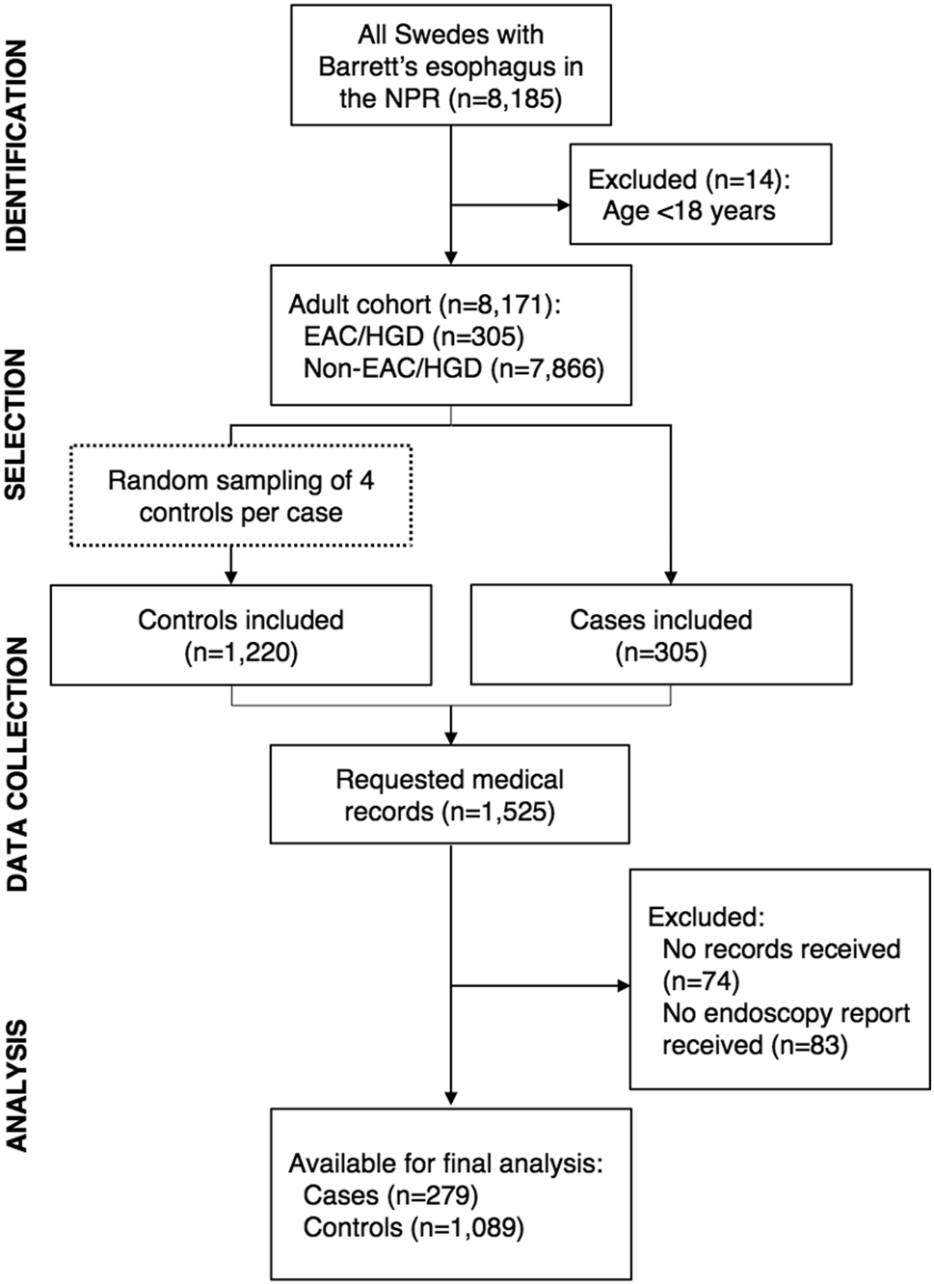
Flowchart describing the selection process from study base to study population. *BE* Barrett’s esophagus, *EAC* esophageal adenocarcinoma, *HGD* high-grade dysplasia, *NPR* National Patient Registry

In total, 305 cases of EAC/HGD and 1220 controls were initially selected from the BE cohort. Among these, complete medical records were retrieved from 71 out of 80 hospitals, rendering 91% (*n* = 279) of cases and 89% (*n* = 1089) of controls for final study participation. Figure 1 shows a flowchart of the selection of cases and controls and Table 1 presents some of their characteristics. Among all participating cases and controls, the mean age at BE diagnosis was 65 years and 71% (*n* = 973) were men. The median maximum BE length was 5.5 cm. A hiatal hernia was described in 80% of all study participants and 55% had a diagnosis of reflux esophagitis. Compared to controls, cases were older, more likely to be male, and had longer maximum and circumferential BE segments (Table 1). While 73% of all EAC cases occurred in long or ultra-long BE segments, 41% of HGD cases occurred in ultra-short or short BE. The vast majority of cases (*n* = 272, 97%) and controls (*n* = 1050, 96%) used proton pump inhibitors. Among the controls, 81% (*n* = 878) of the endoscopies included a biopsy. In these controls, the histopathology report showed specialized intestinal metaplasia in 71% (*n* = 622), fundic or cardiac type metaplasia in 22% (*n* = 193) and no metaplasia (i.e., squamous epithelium) in 4% (*n* = 33). In 4% (*n* = 30), metaplasia was reported but not specified. Furthermore, no dysplasia was present in 79% (*n* = 692), indefinite dysplasia in 1% (*n* = 10), low-grade dysplasia in 16% (*n* = 140), and initially HGD in 1% (*n* = 7), which was subsequently downstaged. The degree of dysplasia was not reported in 3% (*n* = 29). All controls, including non-biopsied, were included in the final analysis, since it was assumed that intestinal metaplasia had been present in previous endoscopies.

**Table 1:** Baseline characteristics of the study participants with esophageal adenocarcinoma (EAC), high-grade dysplasia (HGD) and controls

Characteristic	EAC/HGD	EAC	HGD	Controls
	(*n* = 279)	(*n* = 151)	(*n* = 128)	(*n* = 1,089)
Male sex—number (%)	242 (86.7)	134 (88.7)	108 (84.4)	731 (67.1)
Age—years (mean ± SD)	67.8 ± 10.6	67.5 ± 10.5	68.1 ± 10.8	64.3 ± 12.1
Maximum Barrett length (cm)				
Continuous—median (IQR)	6 (3–10)	6 (4–10)	5 (2–10)	2 (1–5)
Missing—number (%)	36 (12.9)	19 (12.6)	17 (13.2)	216 (19.8)
Categorical—number (%)				
Ultra-short (< 1 cm) or islands	23 (8.2)	10 (6.6)	13 (10.2)	195 (17.9)
Short (1 to < 3 cm)	45 (16.1)	15 (9.9)	30 (23.4)	338 (31.0)
Long (3 to < 8 cm)	97 (34.8)	55 (36.4)	42 (32.8)	302 (27.7)
Ultra-long (≥ 8 cm)	89 (31.9)	55 (36.4)	34 (26.6)	143 (13.1)
Missing	25 (9.0)	16 (10.6)	9 (7.0)	111 (10.2)
Circumferential Barrett length (cm)				
Continuous—median (IQR)	3 (0–8)	4 (0–8)	2 (0–7)	0 (0–2)
Missing—number (%)	88 (31.5)	53 (35.1)	35 (27.3)	326 (30.0)
Categorical—number (%)				
No circumferential lesion	66 (23.7)	30 (19.9)	36 (28.1)	473 (43.4)
< 3 cm	30 (10.8)	11 (7.3)	19 (14.8)	125 (11.5)
3 to < 8 cm	43 (15.4)	28 (18.5)	15 (11.7)	96 (8.8)
≥ 8 cm	52 (18.6)	29 (19.2)	23 (18.0)	69 (6.3)
Missing	88 (31.5)	53 (35.1)	35 (27.3)	326 (30.0)
Presence and size of hiatal hernia (cm)				
Continuous—median (IQR)	3 (2–5)	4 (2–5)	3 (3–5)	3 (2–5)
Missing—number (%)	185 (66.3)	107 (70.9)	78 (60.9)	721 (66.2)
Axial length—number (%)				
No hernia	60 (21.5)	42 (27.8)	18 (14.1)	219 (20.1)
Small (1–2 cm)	46 (16.5)	23 (15.2)	23 (18.0)	235 (21.6)
Medium (> 2 to < 5 cm)	46 (16.5)	15 (9.9)	31 (24.2)	182 (16.7)
Large (≥ 5 cm)	58 (20.8)	35 (23.2)	23 (18.0)	188 (17.3)
Missing	69 (24.7)	36 (23.8)	33 (25.8)	265 (24.3)
Presence of esophagitis—number (%)				
No esophagitis	123 (44.1)	80 (53.0)	43 (33.6)	491 (45.1)
Esophagitis	156 (55.9)	71 (47.0)	85 (66.4)	598 (54.9)

*IQR* Interquartile range, *SD* standard deviation

### Risk factors for tumor progression

Table 2 shows risk estimates for the potential variables age, sex, and endoscopic factors in relation to EAC/HGD combined, as well as EAC and HGD separately, using multiple imputation for missing data. Logistic regression were used to assess the effect of age, sex, BE length, hiatal hernia, and esophagitis as predictors of tumor progression to EAC/HGD. The multivariable analysis showed that older age (OR 1.02 [95% CI 1.01–1.03] per year), male sex (OR 2.8 [95% CI 1.9–4.1]), and increasing maximum BE length at diagnosis (OR 1.1 [95% CI 0.7–1.9] for short segment BE, OR 2.3 [95% CI 1.4–3.9] for long segment BE, and OR 4.3 [95% CI 2.5–7.2] for ultra-long BE, all compared to ultra-short BE) were linearly associated with an increased risk of EAC/HGD. Increasing circumferential extent of the BE segment (Prague C) was also associated with EAC/HGD, but less so than maximum extent (Prague M), and after controlling for Prague M, Prague C was not an independent predictor of EAC/HGD. Presence or size of hiatal hernia was not associated with any of the outcomes. Reflux esophagitis was not associated with EAC/HGD (OR 1.0 [95% CI 0.8–1.4]), but with an increased risk of HGD only (OR 1.6 [95% CI 1.1–2.3]).

**Table 2:** Prediction of EAC/HGD combined, and EAC and HGD separately based on age, sex and endoscopic variables after multiple imputation (including patients with missing data), presented as odds ratio (OR) with 95% confidence interval (CI)

Characteristic	EAC/HGD		EAC		HGD	
	Crude OR	Adjusted OR^a^	Crude OR	Adjusted OR^a^	Crude OR	Adjusted OR^a^
Age (year)						
Continuous	1.03 (1.01–1.04)	1.02 (1.01–1.03)	1.02 (1.01–1.04)	1.02 (1.00–1.03)	1.03 (1.01–1.05)	1.03 (1.01–1.04)
Sex						
Women	1.0 (Reference)	1.0 (Reference)	1.0 (Reference)	1.0 (Reference)	1.0 (Reference)	1.0 (Reference)
Men	3.2 (2.2–4.6)	2.8 (1.9–4.1)	3.9 (2.3–6.5)	3.2 (1.9–5.5)	2.6 (1.6–4.3)	2.4 (1.5–4.0)
Barrett length (cm)						
<1	1.0 (Reference)	1.0 (Reference)	1.0 (Reference)	1.0 (Reference)	1.0 (Reference)	1.0 (Reference)
1 ≤ x < 3	1.2 (0.7–2.0)	1.1 (0.7–1.9)	1.0 (0.4–2.3)	1.0 (0.4–2.3)	1.4 (0.7–2.7)	1.2 (0.6–2.4)
3 ≤ x < 8	2.7 (1.7–4.5)	2.3 (1.4–3.9)	3.7 (1.8–7.3)	3.3 (1.6–6.7)	2.1 (1.1–4.0)	1.6 (0.8–3.1)
≥ 8	5.2 (3.2–8.6)	4.3 (2.5–7.2)	7.5 (3.7–15.3)	6.8 (3.3–14.0)	3.5 (1.8–6.9)	2.6 (1.3–5.1)
Hiatal hernia						
No	1.0 (Reference)	1.0 (Reference)	1.0 (Reference)	1.0 (Reference)	1.0 (Reference)	1.0 (Reference)
Yes	0.9 (0.7–1.3)	0.8 (0.6–1.1)	0.7 (0.4–1.0)	0.6 (0.4–0.9)	1.5 (0.9–2.6)	1.3 (0.8–2.2)
Esophagitis						
No	1.0 (Reference)	1.0 (Reference)	1.0 (Reference)	1.0 (Reference)	1.0 (Reference)	1.0 (Reference)
Yes	1.0 (0.8–1.4)	1.1 (0.8–1.4)	0.7 (0.5–1.0)	0.8 (0.5–1.1)	1.6 (1.1–2.4)	1.6 (1.1–2.3)

^a^Adjusted for all other factors in the table

### Prediction model

**Fig. 2:**
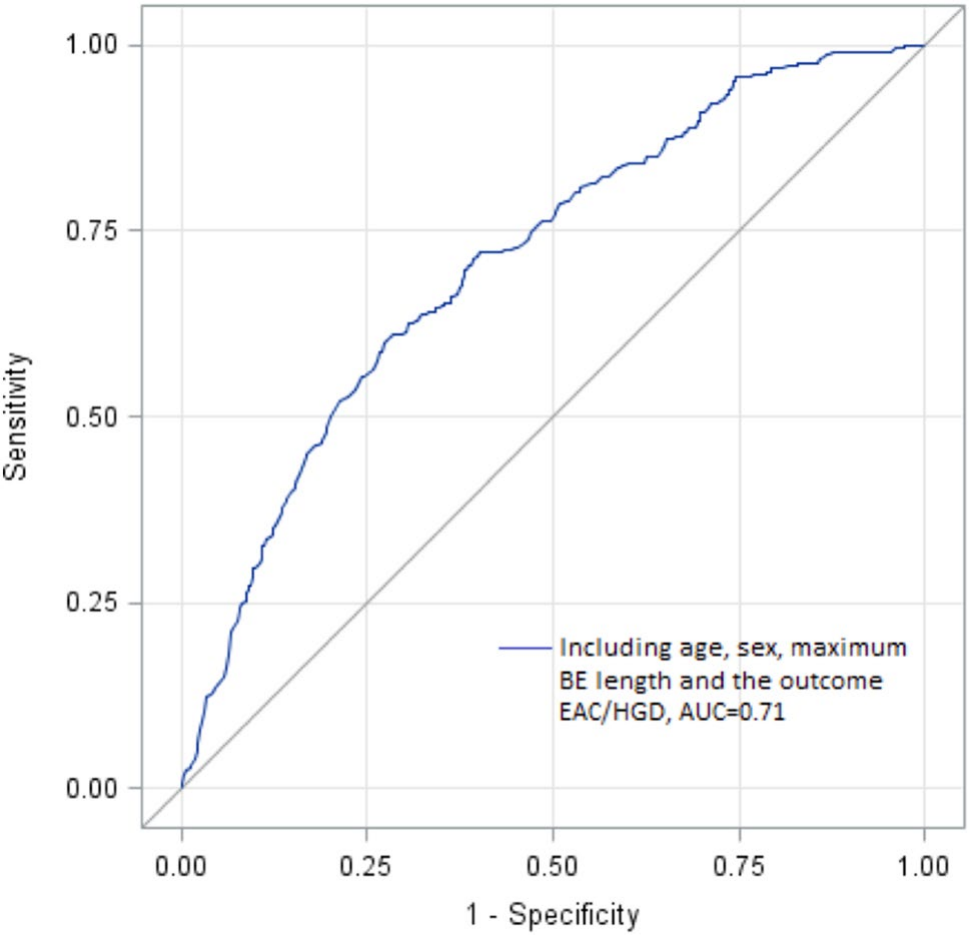
Receiver operating characteristic curve for the final prediction model, based on age, sex, and maximum length of the Barrett’s segment. The area under the curve is 0.71, meaning that 71% of all esophageal adenocarcinoma or high-grade dysplasia can be explained by the model

Based on the results above, the final prediction model incorporated only age, sex, and maximum BE length. This model predicted 71% of all EAC/HGD combined (Fig. 2), 75% of all EAC, and 68% of all HGD cases. The *p*-value for the Hosmer and Lemeshow test was 0.45, confirming the null hypothesis of good fit of the final model. Accuracy statistics, i.e., sensitivity, specificity, false positive rate, and false negative rate are presented for different probability thresholds in Table 3. Additional adjustments for circumferential extent of the BE segment, hiatal hernia, and reflux esophagitis did not alter the coefficients of the included variables and were not associated with EAC/HGD.

**Table 3:** Accuracy statistics of the final prediction model for various probabilities of the main outcome esophageal adenocarcinoma or high-grade dysplasia

Probability threshold	Percentage (%)			
	Sensitivity	Specificity	False positive rate	False negative rate
0.10	95.3	24.9	75.2	4.7
0.15	76.4	47.1	72.7	11.5
0.20	64.6	64.1	68.2	12.6
0.25	59.8	72.3	64.1	12.6
0.30	44.5	82.9	59.6	14.8

### Comparison of analysis strategies

A complete case analysis showed almost identical results as in the multiple imputation analysis (Supplementary Table).

### Effect modification

Effect modification analysis did not show any interactions between any of the three predictors included in the final model (data not shown).

## Discussion

This study indicates that the variables age, sex, and maximum BE length can be combined for good prediction of progression from BE to EAC/HGD, while circumferential extent of the BE, hiatal hernia, and reflux esophagitis does not improve the performance of the prediction model.

This was one of the largest studies on endoscopic factors for tumor progression in BE to date, including almost all cases of EAC/HGD in individuals with a known BE diagnosis in Sweden during a contemporary period. The nationwide and population-based approach with a high participation rate (90%) provided an unselected cohort of individuals with a BE diagnosis from which all cases of EAC/HGD and randomly selected controls were identified, resulting in a low risk of selection bias. The information about predictors and outcomes was based on high-quality registry data and a comprehensive medical record assessment. Additionally, the relatively high number of cases compared to previous studies ensured precise measures of association between the studied risk factors and the main outcome. However, there were also methodological limitations, including limited missing data on the endoscopic variables. Yet, the frequencies of missing data were lower than that in the existing literature [16, 17]. To compensate for the missing data, a multiple imputation analysis was used as the main approach, which showed almost identical results as the complete case analysis, indicating that the imputation worked well. Any influence of potential risk factors not well described in endoscopy report, e.g., obesity and history of smoking, was not examined. Although these factors may improve the model, they were excluded because of the high proportion of missing data and to preserve the internal validity of the study. Another potential limitation is the diagnostic criteria for BE. Some societies require specialized intestinal metaplasia for the diagnosis of BE, while others accept solely fundic or cardiac type metaplasia. While some controls presented without specialized intestinal metaplasia in the studied pathology report, it was presumed that the patients had specialized intestinal metaplasia in previous endoscopies as this is mandatory for the diagnosis of BE in Sweden. Thus, the results from the current study should be generalizable to patients with BE as defined by the presence of specialized intestinal metaplasia.

Risk prediction modeling could be a useful tool for the tailoring of surveillance of individuals with non-dysplastic BE. The finding that the risk of EAC/HGD increases with older age, male sex, and longer BE segment is supported by earlier studies [16–22]. Previous studies, based on demographical data, histopathology data, and biomarkers, have shown comparable accuracy to the model in our study, but were less clinically distinct and applicable [20, 23, 24].

The present study is the first study to create a prediction model based only on age, sex, and BE length. This simple model had a fairly high accuracy to discriminate between cases and controls. It should be highlighted that many early-stage adenocarcinomas arise in short BE segments, indicating that maximum BE length alone is not an ideal predictor of tumor progression [18]. Thus, adding predictive factors other than segment length, i.e., in this model age and sex, is crucial. Studies incorporating other easily available variables, e.g., tobacco smoking, reflux symptoms and use of proton-pump inhibitors, could potentially further improve the selection of absolute high-risk individuals who would benefit from intensified surveillance. In contrast, the presence of obesity and alcohol does not likely improve such a model [25]. The findings of the study may be generalizable to other populations, but this needs to be validated.

Hiatal hernia is an established risk factor for BE, but did not predict further tumor progression in the present study. Some studies have found that the presence of a hiatal hernia in BE accelerates the risk of HGD/EAC [20, 21], but recent prospective studies have not corroborated these results [22, 23], except for a study reporting an increased risk in very large hernias (≥ 6 cm) compared to no hernia [26]. The present study found no role of hiatal hernia of any size in the progression from BE to EAC/HGD, which is in line with more recent studies.

As with hiatal hernia, reflux esophagitis is a risk factor for BE, but the carcinogenic role of reflux esophagitis in the BE lesion is not established [27]. One study suggested that esophagitis on index endoscopy is associated with tumor progression in BE, but did not show any increasing risk with more severe grades of esophagitis [23]. To our knowledge, the present study is the largest assessing reflux esophagitis as a risk factor of EAC/HGD in individuals with BE. The results showed an increased risk of HGD, but not EAC/HGD or EAC alone. This latter finding needs to be interpreted cautiously, however, because the assessment of dysplasia is notoriously unreliable in individuals with BE mucosa with concomitant esophagitis.

As mentioned above, BE patients enrolled in surveillance programs are diagnosed with earlier stage EAC and probably survive longer than those diagnosed with EAC outside surveillance programs [28–32]. To increase the efficiency of surveillance, some gastroenterological societies now recommend that BE patients with maximum BE length > 3 cm undergo endoscopy more often [33], while some recommend surveillance only in those with additional risk factors for EAC, such as older age, male sex, and obesity [34]. The results from our study corroborates that individuals with maximum BE length > 3 cm should be monitored more frequently, and that individuals at old age, particularly males, may benefit from intensified surveillance independent of the BE segment length, while younger individuals likely do not need surveillance until they reach a certain age.

In conclusion, this nationwide Swedish study indicates that a prediction model including only age, sex, and maximum length of the BE segment has good discriminative accuracy for progression to EAC/HGD, while circumferential extent of the BE, hiatal hernia, and esophagitis did not improve the model. If these results are confirmed in future studies of other populations, this clinical prediction model may contribute to a more individually tailored surveillance of people diagnosed with BE.

## Electronic supplementary material

Below is the link to the electronic supplementary material.

Supplementary material 1 (DOCX 15 KB)
