# Supplementary material for: Clinical prediction model for tumor progression in Barrett’s esophagus
Source: Surg Endosc. 2018 Nov 19;33(9):2901–8. doi: 10.1007/s00464-018-6590-5 (PMC6684532; doi:10.1007/s00464-018-6590-5)
Supplement: Supplementary file 1 — Supplementary material 1 (DOCX 15 KB) [file 464_2018_6590_MOESM1_ESM.docx]

**Supplementary Table. Prediction of esophageal adenocarcinoma (EAC)/high-grade dysplasia (HGD) combined, and EAC and HGD separately based on age, sex and endoscopic variables in a complete case analysis (excluding patients with missing data), presented as odds ratio (OR) with 95% confidence interval (CI)**

| **Characteristic** | **EAC/HGD** | | **EAC** | | **HGD** | |
| --- | --- | --- | --- | --- | --- | --- |
|  | Crude OR | Adjusted OR* | Crude OR | Adjusted OR* | Crude OR | Adjusted OR* |
| **Age (year)** | | | | | | |
| Continuous | 1.03 (1.01 – 1.04) | 1.02 (1.01 – 1.03) | 1.02 (1.01 – 1.04) | 1.01 (1.0 – 1.03) | 1.03 (1.01 – 1.05) | 1.03 (1.01 – 1.05) |
| **Sex** | | | | | | |
| Women | 1.0 (Reference) | 1.0 (Reference) | 1.0 (Reference) | 1.0 (Reference) | 1.0 (Reference) | 1.0 (Reference) |
| Men | 3.3 (2.2 – 4.8) | 2.8 (1.9 – 4.2) | 4.2 (2.4 – 7.4) | 3.5 (2.0 – 6.3) | 2.6 (1.5 – 4.7) | 2.3 (1.4 – 3.9) |
| **Barrett length (cm)** | | | | | | |
| <1 | 1.0 (Reference) | 1.0 (Reference) | 1.0 (Reference) | 1.0 (Reference) | 1.0 (Reference) | 1.0 (Reference) |
| 1≤x<3 | 1.1 (0.7 – 1.9) | 1.1 (0.6 – 1.8) | 0.9 (0.4 – 2.0) | 0.8 (0.4 – 1.9) | 1.3 (0.7 – 2-6) | 1.2 (0.6 – 2.3) |
| 3≤x<8 | 2.7 (1.7 – 4.4) | 2.3 (1.4 – 3.7) | 3.6 (1.8 – 7.1) | 3.1 (1.5 – 6.3) | 2.1 (1.1 – 4.0) | 1.6 (0.8 – 3.1) |
| ≥8 | 5.3 (3.2 – 8.8) | 4.2 (2.5 – 7.0) | 7.5 (3.7 – 15.2) | 6.4 (3.1 – 13.3) | 3.6 (1.8 – 7.0) | 2.6 (1.3 – 5.1) |
| **Hiatal hernia** | | | | | | |
| No | 1.0 (Reference) | 1.0 (Reference) | 1.0 (Reference) | 1.0 (Reference) | 1.0 (Reference) | 1.0 (Reference) |
| Yes | 1.1 (0.8 – 1.6) | 1.0 (0.8 – 1.5) | 0.9 (0.6 – 1.3) | 0.7 (0.4 – 1.1) | 1.7 (1.0 – 3.0) | 1.4 (0.8 – 2.5) |
| **Esophagitis** | | | | | | |
| No | 1.0 (Reference) | 1.0 (Reference) | 1.0 (Reference) | 1.0 (Reference) | 1.0 (Reference) | 1.0 (Reference) |
| Yes | 1.1 (0.8 – 1.5) | 1.1 (0.8 – 1.5) | 0.8 (0.6 – 1.2) | 0.8 (0.6 – 1.2) | 1.6 (1.1 – 2.4) | 1.6 (1.0 – 2.4) |
